# Evidence-Based Conservative Treatment Strategies for Lumbar Radiculopathy: A Systematic Review

**DOI:** 10.7759/cureus.110554

**Published:** 2026-06-09

**Authors:** Aishwarya P Shinde, Akshaya V Joshi, Sandeep B Shinde, Mebin S Thomas

**Affiliations:** 1 Department of Musculoskeletal Sciences, Krishna College of Physiotherapy, Krishna Vishwa Vidyapeeth (Deemed to be University), Karad, IND

**Keywords:** conservative management, degeneration, neural tissue mobilization, spine disease, straight leg raise

## Abstract

Lumbar radiculopathy is a prevalent and debilitating spinal condition that causes severe pain, functional impairment, and diminished quality of life. Although conservative treatments are widely recommended as first-line management, establishing their comparative effectiveness remains challenging due to substantial heterogeneity in the literature. This systematic review synthesized and critically appraised the evidence evaluating non-surgical, conservative interventions for lumbar radiculopathy. Adhering to the Preferred Reporting Items for Systematic Reviews and Meta-Analyses (PRISMA) 2020 guidelines, this review was prospectively registered with PROSPERO (CRD420261407070). Comprehensive electronic searches were conducted across PubMed, Scopus, Web of Science, Embase, and the Cochrane Library up to 2026. Randomized controlled trials (RCTs) involving participants with clinically or radiologically confirmed lumbar radiculopathy were eligible for inclusion. Methodological quality was evaluated using the Cochrane Risk of Bias 2 (RoB 2) tool. Due to marked clinical and methodological heterogeneity, a quantitative meta-analysis was precluded, and a structured narrative synthesis was performed instead.

Nineteen RCTs published between 2016 and 2026 met the inclusion criteria. The investigated therapies included manual therapy, neural mobilization, exercise rehabilitation, physical modalities, and multimodal regimens. Rather than purely qualitative benefits, individual trials demonstrated quantifiable short-term improvements. Manual therapy combined with exercise or neurodynamics yielded significant advantages over conventional care; for instance, adding spinal mobilization with leg movement (SMWLM) achieved a mean difference (MD) in leg pain reduction of 2.0 (95% CI: 1.4 to 2.6) at two weeks and 2.6 (95% CI: 1.9 to 3.2) at six months. Similarly, targeted neural mobilization outperformed traditional therapy at eight weeks (pain MD: -2.4, 95% CI: -3.1 to -1.7; disability MD: -12.8%, 95% CI: -16.1% to -9.5%). Traditional Persian manual therapy combined with exercise reduced low back pain by 4.28 units (95% CI: 3.36 to 5.19). However, clinical superiority was inconsistent across all outcome domains, long-term follow-ups were rare, and conservative treatment non-response or failure remained a distinct clinical possibility. Methodological limitations, including a high risk of bias regarding incomplete blinding of participants/therapists and selective outcome reporting, were frequently observed across multiple trials.

Conservative interventions yield measurable, statistically significant short-term improvements in pain and function for lumbar radiculopathy. However, the overall certainty of the evidence is limited by high clinical heterogeneity and trial-level methodological constraints, which precluded data pooling. Findings should be interpreted cautiously, and further high-quality trials with standardized outcomes and adverse event reporting are required.

## Introduction and background

Epidemiology and clinical burden

Lumbar radiculopathy is a common and clinically significant spinal disorder characterized by pain radiating from the lower back into the lower extremity due to compression or irritation of one or more lumbar nerve roots. It is most frequently connected with lumbar disc herniation, degenerative spinal changes, or mechanical dysfunctions affecting the intervertebral foramen [1].

The burden of lumbar radiculopathy is substantial, contributing to pain-related disability, reduced functional capacity, and diminished quality of life across working-age and older populations. Conservative management is widely recommended as the first-line approach, especially in the absence of red flags or progressive neurological deficits. Evidence-based guidelines emphasize non-surgical interventions aimed at pain reduction, neural decompression, and restoration of functional movement patterns [2].

Pathophysiology and diagnosis

The underlying pathology of lumbar radiculopathy centers on the compression, inflammation, or irritation of one or more specific lumbar nerve roots. These structural changes are typically secondary to underlying pathologies such as disc herniations or localized intervertebral foramen obstructions, which ultimately alter neural mechanics and increase nerve sensitivity [1]. Clinically, lumbar radiculopathy is diagnosed based on a combination of characteristic dermatomal leg pain, neurological deficits such as altered sensation, reduced muscle strength, or diminished reflexes, and confirmatory imaging findings, particularly magnetic resonance imaging (MRI), that demonstrate nerve root involvement consistent with the patient’s symptoms [3].

Current conservative management approaches

Manual therapy has evolved as a cornerstone of conservative care for lumbar radiculopathy. Techniques such as spinal manipulation, mobilization, and neural mobilization are frequently used to address mechanical and neurophysiological contributors to radicular pain. Randomized controlled trials (RCTs) have reported significant improvements in pain intensity and functional disability following manual spinal mobilization-based interventions in patients with lumbar radiculopathy [3,4]. Similarly, mobilization-based approaches, including spinal mobilization with leg movement (SMWLM), have shown significant improvements in pain and functional outcomes compared with control or conventional care, particularly in patients presenting with positive neurodynamic tests and radiating leg symptoms [5].

Neural mobilization techniques target altered neural mechanosensitivity and impaired nerve excursion, which are commonly observed in lumbar radiculopathy. Comparative trials have indicated that neural mobilization may be as effective as, or in some cases superior to, traditional manual therapy approaches in improving pain and functional disability in patients diagnosed with lumbar radiculopathy using clinical neurological signs and imaging evidence [6]. Shacklock’s neural mobilization, in particular, has demonstrated beneficial effects in acute and subacute lumbar disc prolapse with radiculopathy, highlighting its relevance across different stages of the condition [1-4].

Exercise-based interventions also play a vital role in comprehensive management strategies. Exercise-based rehabilitation combined with manual spinal mobilization has been shown to reduce pain intensity and functional disability in patients with lumbar radiculopathy [3]. These findings support the concept that addressing proximal stability and regional mobility may influence neural loading and symptom modulation.

Recent trials have explored multimodal treatment strategies, combining manual therapy with neurodynamic techniques. Evidence suggests that spinal manipulation or mobilization used as an adjunct to neurodynamic mobilization may produce better results than with isolated interventions in patients with lumbar disc herniation and radiculopathy confirmed by clinical and radiological criteria [7]. These findings underscore the importance of integrated treatment approaches tailored to the multifactorial nature of lumbar radiculopathy.

Adjunctive physical modalities have further expanded conservative treatment options. Low-level laser therapy has demonstrated effectiveness in patients with discogenic lumbar radiculopathy confirmed by imaging, showing significant reductions in pain and disability compared with sham or conventional interventions [8]. Spinal decompression therapy, when added to routine physical therapy, has also been reported to improve pain, spinal mobility, endurance, and quality of life in individuals with clinically diagnosed lumbar radiculopathy [2-5].

Spinal manipulation techniques continue to be refined, with studies comparing different manipulative approaches in acute radiculopathy caused by lumbar disc herniation. This study demonstrates evidence-based interventions emphasizing effectiveness have careful patient selection based on clinical diagnosis, neurological examination, and imaging confirmation to balance therapeutic benefits against potential risks. Additionally, positional distraction techniques combined with stabilization exercises have shown promise in reducing pain and disability compared with stabilization exercises alone in patients with clinically diagnosed lumbar radiculopathy [3-6].

Gaps in existing evidence

Despite the growing volume of RCTs and protocols, variability remains in diagnostic criteria, intervention selection, and outcome reporting across studies. Most trials rely on a combination of clinical examination such as dermatomal pain distribution, positive straight leg raise (SLR) or slump tests, neurological deficits and imaging confirmation to establish a diagnosis of lumbar radiculopathy [1]. However, differences in chronicity, underlying pathology, and intervention dosage complicate direct comparisons across studies.

Aims and objectives of this review

This systematic review aims to critically appraise and integrate current evidence on conservative, evidence-based interventions for the treatment of lumbar radiculopathy. The review focuses on identifying and classifying commonly applied conservative management approaches such as physiotherapy, exercise-based programs, manual therapy techniques, neural mobilization, electrotherapeutic modalities, and patient education and evaluating their effectiveness in pain reduction, functional improvement, resolution of neurological symptoms, and enhancement of quality of life. Furthermore, this review assesses the methodological rigor and overall strength of evidence of the included studies, with particular emphasis on randomized controlled trials and high-quality observational research. Comparative analysis of outcomes across different conservative treatment modalities is undertaken, and existing gaps in the literature are highlighted. The findings are intended to inform evidence-based clinical practice, optimize conservative care pathways, and potentially reduce the reliance on surgical interventions for individuals with lumbar radiculopathy.

## Review

Methodology

Study Design

In accordance with the Preferred Reporting Items for Systematic Reviews and Meta-Analyses (PRISMA) statement, this review implemented standardized protocols for data collection and reporting to guarantee scientific transparency [9]. The systematic review was registered with PROSPERO (Registration No. CRD420261407070; registered on 27 may 2026).

Search Strategy

To ensure a thorough evidence synthesis, systematic searches were conducted in PubMed, Web of Science, Scopus, Cochrane, and Embase from 2016 to 2026. The full, unabridged Boolean search strings utilized for all databases are provided in the Appendix to ensure complete replicability. The strategy incorporated a mix of MeSH terminology and specific keywords ranging from “lumbar radiculopathy” to “clinical special tests,” integrated via Boolean operators. This approach allowed for a targeted yet broad exploration of both clinical and radiographic assessment methods. In addition to the electronic database queries, manual hand-searching (citation chaining) of the reference lists of all included full-text articles and previously published reviews was conducted to identify any relevant studies not captured by the initial algorithmic search.

Inclusion Criteria

To be eligible for inclusion, studies were required to be published in English and involve human participants of any age or sex with a confirmed diagnosis of lumbar radiculopathy. The diagnostic framework had to be clearly defined through clinical assessment, diagnostic testing, or validated criteria. Furthermore, eligibility was restricted to RCTs involving patients whose radicular symptoms were consistent with nerve root involvement and corroborated by clinical findings, imaging, or neurophysiological evaluation.

Exclusion Criteria

Studies were excluded if they were published in languages other than English or utilized animal or cadaveric models. The review also omitted investigations primarily focused on artificial intelligence or machine learning-driven diagnostic algorithms. Furthermore, including case reports, case series, editorials, narrative reviews, and conference abstracts lacking full-text availability, was excluded. Finally, research involving paediatric cohorts, postoperative spinal conditions, or exclusively surgical interventions with no application to clinical diagnosis or physiotherapy-based conservative care was deemed ineligible.

**Figure 1:**
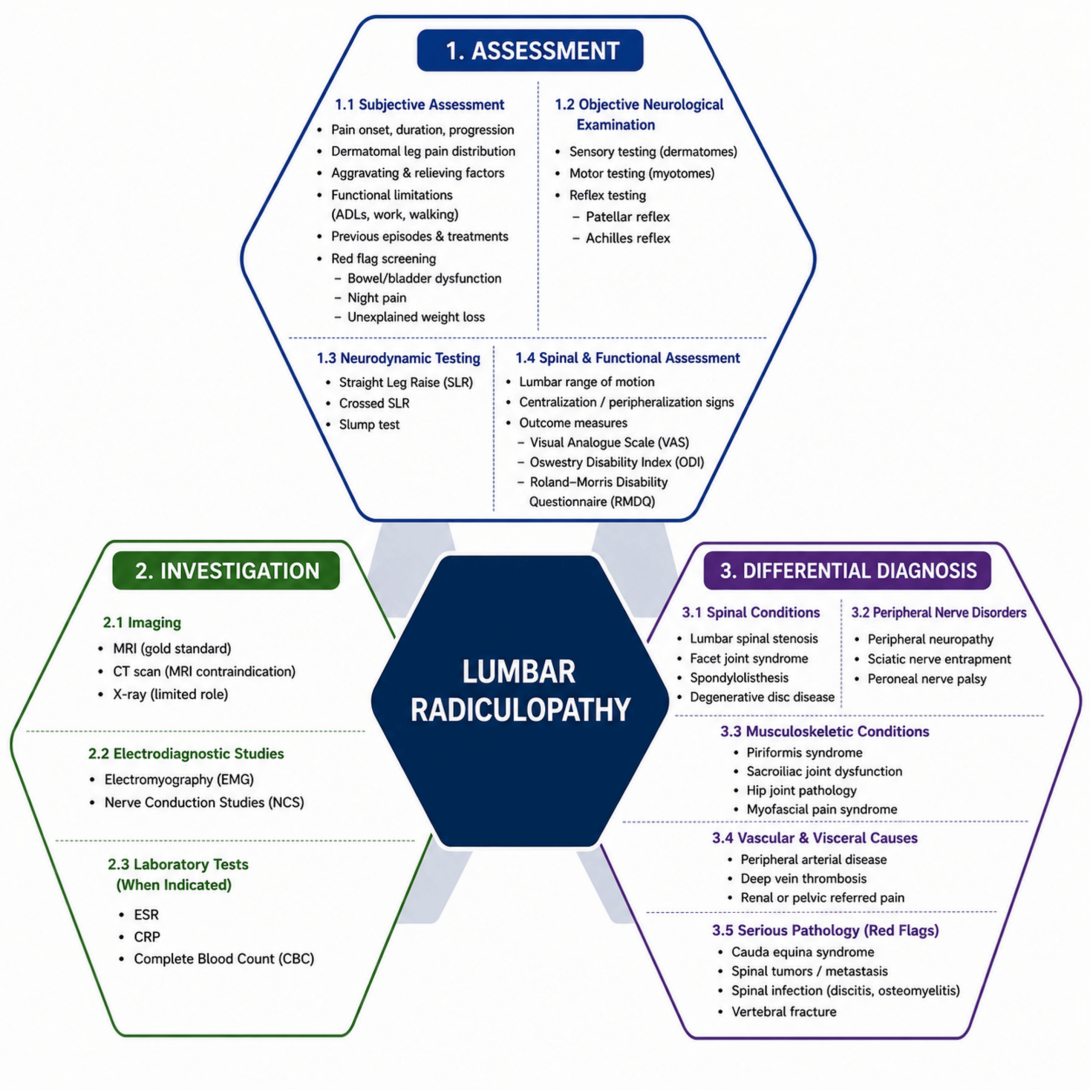
Comprehensive clinical framework for lumbar radiculopathy, outlining assessment components, diagnostic investigations, and differential diagnosis to support accurate clinical decision-making. ADLs: activities of daily living; ESR: erythrocyte sedimentation rate; CRP: C-reactive protein

Figure 1 illustrates a comprehensive clinical framework for lumbar radiculopathy, outlining assessment components, diagnostic investigations, and differential diagnosis to support accurate clinical decision-making [10].

Study Selection and Data Extraction 

Following PRISMA recommendations, two reviewers independently screened all identified articles against the inclusion criteria. Discrepancies were resolved by discussion and consensus, with involvement of a third reviewer when required. Inter-rater reliability was quantified using Cohen’s kappa [9].

Quality Assessment 

To account for the heterogeneity of the research designs, methodological quality and risk of bias were evaluated using validated, study-specific instrument. Specifically, the Cochrane Collaboration’s Risk of Bias (RoB 2) tool was employed to rigorously assess the diagnostic reliability and internal validity of the included RCTs [11].

Data Synthesis

Due to substantial clinical and methodological heterogeneity among the included studies, particularly in diagnostic approaches ranging from manual provocative tests to dynamic imaging modalities, as well as variability in interventions and outcome measures, a quantitative meta-analysis was not appropriate. This approach is consistent with the recommendations of the PRISMA 2020 statement, which supports narrative synthesis when statistical pooling is unjustified. Accordingly, a structured narrative synthesis was undertaken in line with the Synthesis Without Meta-analysis (SWiM) reporting guideline, providing a transparent framework for synthesizing heterogeneous evidence. Extracted data were systematically categorized and analysed across two predefined clinical domains: individual clinical examinations and clustered special tests, and dynamic imaging and kinematic assessments, ensuring clarity, transparency, and reproducibility of the synthesis.

Results

**Figure 2:**
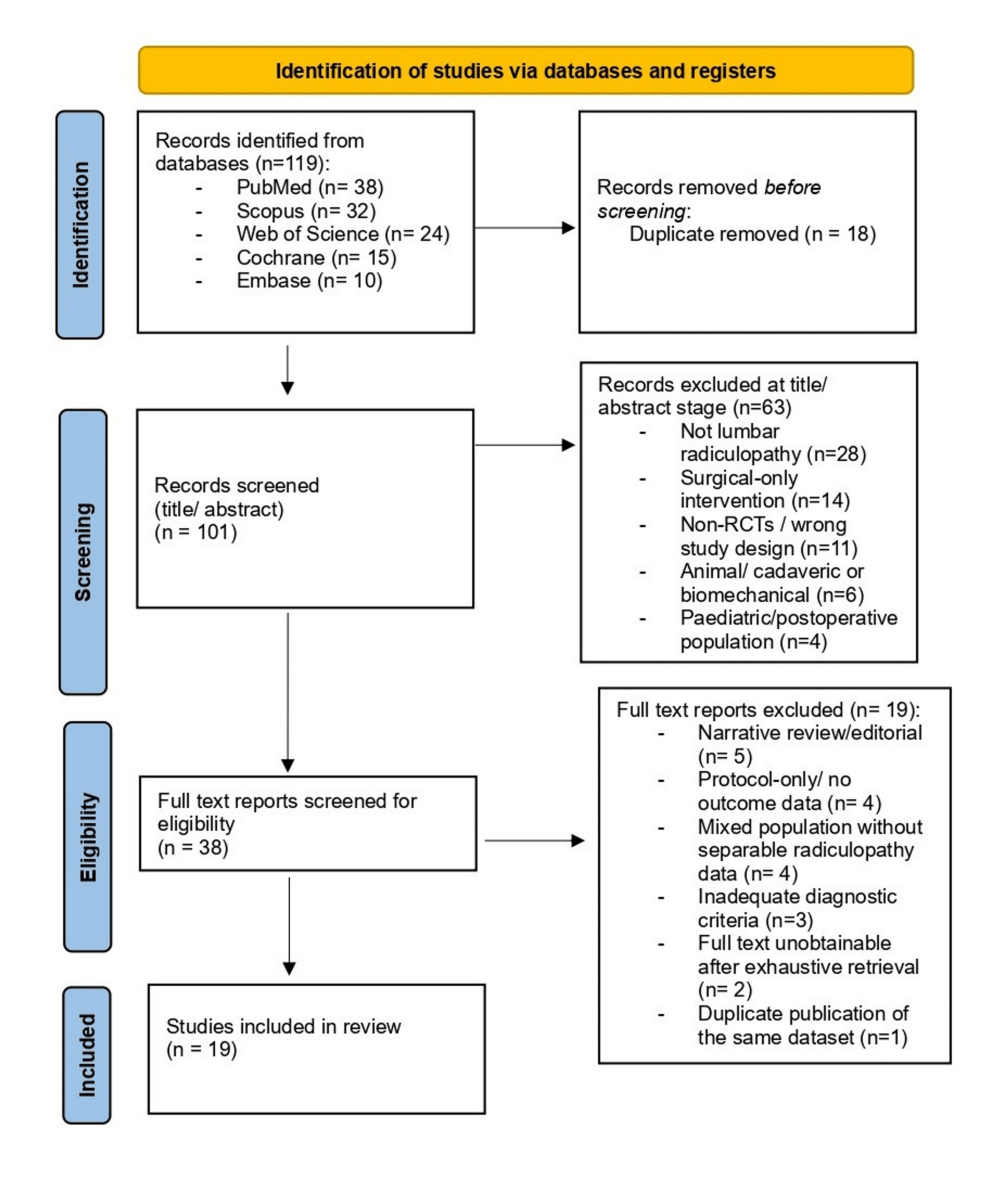
PRISMA flowchart PRISMA: Preferred Reporting Items for Systematic Reviews and Meta-Analysis; RCTs: randomized controlled trials

Figure 2 shows the PRISMA flow chart [9]. 

A total of 19 clinical trials published from 2016 to 2026 were included in this review, primarily RCTs evaluating interventions for lumbar radiculopathy or radicular low back pain. Participants were adults, with mean ages generally ranging from approximately 40 to 60 years, reflecting a predominantly middle-aged population. Most studies enrolled patients with chronic symptoms, while fewer included subacute or acute cases. Diagnosis was typically based on clinical examination supported by imaging, most commonly MRI-confirmed lumbar disc herniation, although diagnostic criteria varied across studies. The trials were conducted across India, Asia, Europe, Africa, and the Middle East, reflecting diverse clinical settings. Interventions investigated included manual therapy techniques, neural and neurodynamic mobilization, exercise-based rehabilitation programs, physical modalities, and combined multimodal approaches, usually compared with conventional physiotherapy. Primary outcomes focused on pain intensity and functional disability, with secondary outcomes such as range of motion (ROM) and quality of life, assessed over short- to long-term follow-up periods. Initial screening of the 119 records demonstrated substantial inter-reviewer agreement (Cohen’s κ = 0.81). Overall, the evidence suggests these interventions are effective in reducing pain and improving function, particularly when manual therapy is combined with exercise or neural mobilization, although methodological and clinical heterogeneity limits direct comparisons across studies.

The details of the 19 included article are summarized in Table 1.

**Table 1:** Summary of 19 randomized controlled trials evaluating conservative interventions for lumbar radiculopathy. VAS: Visual Analogue Scale; QoL: quality of life; SLR: straight leg raise; PINS: posteroanterior intermittent nucleus mobilization; SMWLM: spinal mobilization with leg movement; ROM: range of motion; ODI: Oswestry Disability Index; SBI: Sciatica Bothersomeness Index; NSD: non-surgical spinal decompression; TUG: Time Up and Go Test; RMDQ: Roland Morris Disability Questionnaire; NDI: Neck Disability Index; DPQ: Dallas Pain Questionnaire; CPT: conventional physical therapy; GROC: Global Rating of Change; MD: mean difference; PPT: pressure pain thresholds; NPRS: Numerical Pain Rating Scale; FTF: finger-to-floor; TFESI: transforaminal epidural steroid injection; CESI: caudal epidural steroid injection; MMST: Modified-Modified Schober’s Test; BME: back muscle endurance; SF-36: Short Form 36; TENS: transcutaneous electrical nerve stimulation; EQ-5D: EuroQol 5-Dimension; PASE: Physical Activity Scale for the Elderly; NMT: neural mobilization techniques; MOLBPQ: Modified Oswestry Low Back Pain Questionnaire; CI: coefficient interval; MCID: minimal clinically important difference; S-LANSS: Self-Administered Leeds Assessment of Neuropathic Symptoms and Signs

Study Title	Author(s), Year	Total Participants	Outcome Measures	Intervention (Study Groups)	Follow-up Duration	Results (p-value)	Conclusion
Comparative effectiveness of 2 manual therapy techniques in lumbar radiculopathy: a randomized control trial [12]	Bello et al., 2019	40	Primary: Pain intensity (measured via the VAS) and functional disability (measured via the Roland-Morris Disability Questionnaire). Secondary: QOL (SF-36 Health Survey), SBI, sciatica frequency (Sciatica Frequency Index), and general perception of recovery (GROC Scale).	Group 1: Received PINS. Group 2: Received SMWLM.	Evaluated at baseline (0 weeks), mid-intervention (4 weeks), and post-intervention (8 weeks).	Both groups demonstrated statistically significant improvements (P < .001) within their respective groups across all primary and secondary outcome measures over time. However, there were no statistically significant differences (P > .05) found between the two groups for any of the measured outcomes at either the 4-week or 8-week marks.	Both manual therapy techniques (PINS and SMWLM), when combined with stabilization and stretching exercises, are effective at reducing pain and disability for individuals with lumbar radiculopathy, but neither technique demonstrated clinical superiority over the other.
The effect of spinal mobilization with leg movement with lumbar radiculopathy: a double blind randomised radiculopathy [13]	Satpute et al., 2019	60	Primary outcomes: Leg pain intensity and functional disability via the ODI score. Secondary outcomes: Low back pain intensity, GROC scale, SLR ROM, and lumbar ROM.	Experimental Group (n=30): Received SMWLM combined with conventional exercise and electrotherapy. Control Group (n=30): Received conventional exercise and electrotherapy alone.	Evaluations were conducted blindly at baseline, immediately post-intervention (2 weeks), at 3 months, and at 6 months.	While both groups demonstrated clinical improvements over time, the experimental group achieved significantly greater benefits. At 2 weeks: The SMWLM group showed a greater reduction in leg pain (Mean Difference [MD] = 2.0; 95% CI, 1.4–2.6) and functional disability (MD = 3.9; 95% CI, 5.5–2.2) compared to the control group. At 6 months: The distinct advantages of SMWLM persisted long-term, showing superior improvements in leg pain (MD = 2.6; 95% CI, 1.9–3.2) and disability (MD = 4.7; 95% CI, 6.3–3.1). The SMWLM cohort also achieved better overall outcomes in patient satisfaction (GROC) and physical SLR mobility.	In individuals dealing with lumbar radiculopathy, adding SMWLM to a standard exercise and electrotherapy regimen yields significantly improved, clinically meaningful benefits for back/leg pain, disability, physical ROM, and patient satisfaction in both the short and long term.
Motor control training compared with transcutaneous electrical nerve stimulation in patients with disc herniation with associated radiculopathy: a randomized controlled trial [14]	França FJ et al., 2019	90	Pain Intensity: NPRS Nerve Root Mobility/Tension: SLR measured via goniometry Functional Disability: MOLBPQ.	Participants were randomly allocated into three equal groups (n = 30 each): 1. Control Group: Conventional physiotherapy (back extension exercises) and hot packs. 2. Experimental Group 1: Shacklock's neural tissue mobilization plus conventional physiotherapy 3. Experimental Group 2: Mulligan's SMWLM combined with neural mobilization and conventional physiotherapy.	Very short-term. Assessments were gathered at three milestones: baseline, immediately post-treatment, and at a follow-up window 48 to 72 hours post-intervention.	Intra-group improvement: All three tracking groups achieved statistically significant improvements across all metrics over the 6 weeks (p < 0.05). Inter-group comparison: Experimental Group 2 (SMWLM + Neural Mobilization + Conventional Physiotherapy) demonstrated a significantly higher mean change and superior paired t-test metrics compared to both Experimental Group 1 and the Control Group at the 6-week final reading (p = 0.000).	Motor control training is significantly more effective than TENS alone for relieving pain, reducing functional disability, improving pain quality, and enhancing core stabilizer (transversus abdominis) activation capacity in patients suffering from lumbar disc herniation with radiculopathy.
Effects of adding a neurodynamic mobilization to motor control training in patients with lumbar radiculopathy due to disc herniation a randomized clinical trial [15]	Plaza-Manzano et al., 2020	32	Primary outcomes: Pain intensity and related functional disability. Secondary outcomes: Neuropathic symptoms, SLR, ROM, mechanical sensitivity, and PPT.	Experimental Group (n=16): Received neurodynamic mobilization (neural gliding/sliding techniques) combined with a motor control exercise program. Control Group (n=16): Received the motor control exercise program alone.	Evaluated at four distinct intervals: baseline (0 weeks), mid-treatment (after 4 visits), post-treatment (after 8 visits), and a long-term follow-up at 2 months.	Both groups achieved similar and statistically large improvements in overall pain intensity, functional disability, and pressure pain thresholds over time. No statistically significant between-group differences (P > .05) were found for these specific markers. However, the group that received the additional neurodynamic mobilization experienced significantly greater improvements (P < .01) specifically in neuropathic symptom reduction and increased SLR mobility compared to those who only performed motor control training.	Incorporating neurodynamic mobilization into a motor control exercise routine provides distinct, targeted benefits by accelerating the clearance of neuropathic symptoms and lowering neural mechanical sensitivity (improving SLR range) in patients with lumbar radiculopathy due to disc herniation. However, it does not provide an additive advantage over a standalone motor control program for generalized back pain or overall functional disability.
Efficacy of the lumbar stabilization and thoracic mobilization exercise program on pain intensity and functional disability reduction in chronic low back pain patients with lumbar radiculopathy: a randomized controlled trial [16]	Kostadinović et al., 2020	80	Pain intensity (assessed for both the lumbosacral spine and the radiating leg via a 100mm VAS). Functional disability reduction.	All participants received a baseline passive physiotherapy protocol consisting of laser therapy and transcutaneous electrical nerve stimulation, paired with an 8-week kinesiotherapy program (divided into three progressive phases based on painless range of motion). They were randomized 1:1 into two structural exercise streams: Experimental Group (n=40): Performed lumbar stabilization exercises combined with targeted thoracic mobilization exercises in a closed kinetic chain (utilizing exercises like the "cat-camel"). Control Group (n=40): Performed standard lumbar stabilization exercises alone, alternating between open and closed kinetic chains.	Evaluated at three distinct intervals: baseline (0 weeks), mid-intervention (4 weeks), and immediately post-intervention (8 weeks).	Both cohorts displayed progressive, statistically significant intra-group improvements (P < 0.05) from baseline across all tracking points. However, the experimental group achieved statistically superior improvements (P < 0.05) compared to the control group at both the 4-week and 8-week evaluations regarding pain relief (back and leg) and functional disability scores.	Integrating thoracic mobilization techniques in a closed kinetic chain alongside deep core lumbar stabilization exercises yields a significantly more effective clinical recovery pathway for patients with chronic low back pain and radiculopathy than standard lumbar stabilization programs alone.
Persian manual therapy method for chronic low-back pain with lumbar radiculopathy: a randomized controlled trial [17]	Sanei et al., 2020	48	Primary outcome: Functional disability measured via the RMDQ. Secondary outcomes: Pain intensity parameters measured via individual VAS for general low back pain, radicular radiating pain, and lower extremity paresthesia (numbness/tingling). Physical lumbar flexibility and tissue excursion were assessed using the FTF test.	Experimental Group (n=24 analyzed): Received a 16-minute traditional Persian manual soft tissue manipulation (known clinically as the Fateh technique or Ghamz therapy) administered once a week for 4 weeks. This localized protocol involves specialized manual steps including deep tissue thumb/finger frictions, rotary palmar pressure, deep petrissage across the gluteal structures, sub-popliteal deep petrissage, and targeted calf manipulations. This group also performed two daily active home exercises. Control Group (n=24 analyzed): Performed the two daily active home exercises alone (standing leg lifts and knee-to-chest stretches) for 4 weeks without any manual therapy session.	Measurements taken at baseline (0 weeks), mid-study post-treatment completion at 4 weeks, and long-term at 8 weeks (4 weeks after all interventions had ceased).	Both cohorts showed improvements, but the group receiving the Persian manual therapy achieved significantly greater therapeutic changes (P < .05) across every tracked category. The Fateh technique intervention significantly decreased: Low back pain by 4.28 units [95% CI, 3.36 to 5.19] Radiculopathy symptoms by 3.85 units [95% CI, 2.67 to 5.03] Paresthesia presence by 1.32 units [95% CI, 0.37 to 2.27] Disability scores by 4.58 units [95% CI, 3.23 to 5.93] It also increased physical forward trunk flexibility by an average of 35.42 mm on the finger-to-floor test. No clinical adverse events were reported.	The traditional Persian manual tissue manipulation (Fateh technique) is a safe, structurally stable, and clinically effective conservative treatment choice. When paired with basic active stretching, it provides superior short- and long-term relief from pain, nerve root irritation, paresthesia, and functional restriction compared to utilizing a standalone home exercise plan.
Effect of supraneural transforaminal epidural steroid injection combined with caudal epidural steroid injection with catheter in chronic radicular pain management: double blinded randomized controlled trial [18]	Munjupong S & Kumnerddee, 2020	54	Pain relief: Measured via the Verbal Numerical Rating Scale. An "effective response" to pain treatment was predefined as a reduction of at least 30% from baseline. Functional outcome: Evaluated using the ODI. A clinically successful functional outcome was predefined as an improvement of at least 15 points from baseline.	Participants were randomly allocated into one of two parallel groups: TC Group (Combined, n=27): Received a supraneural TFESI combined with a CESI via a catheter. T Group (Standalone, n=27): Received a supraneural TFESI alone.	Follow-up duration: Evaluated blindly by a single assessor at baseline (pre-procedure), 1 month, 3 months, and 6 months post-procedure.	Both treatment groups demonstrated a statistically significant reduction in average Verbal Numerical Rating Scale pain scores from baseline across all follow-up intervals (1, 3, and 6 months; P < .05). Between-group (Pain): The combination (TC) group exhibited significantly more effective pain relief compared to the standalone TFESI (T) group at the 3-month mark (P = .01). However, subgroup analysis showed no distinct statistical differences in long-term pain relief at 6 months. Between-group (Function): There were no statistically significant differences between the two groups regarding functional disability improvements (ODI scores) at any of the follow-up periods.	Combining a supraneural TFESI with a CESI via catheter provides significantly better mid-term pain relief (at 3 months) than a transforaminal injection alone for chronic lumbosacral radicular pain. However, this dual approach does not provide any additional clinical benefit regarding long-term functional recovery or disability reduction.
Spinal manipulation for subacute and chronic lumbar radiculopathy: a randomized controlled trial [19]	Ghasabmahaleh et al., 2021	44	Primary outcome: Intensity of low back pain measured on a VAS. Secondary outcomes: Functional status measured via the ODI score, spinal ROM, and the SLR. Timeline: Assessments were taken at baseline, immediately post-intervention, and at a 3-month follow-up.	Both groups: Received a baseline regimen of standard physical therapy (physiotherapy). Manipulation (Experimental) Group: Received three sessions of spinal manipulation therapy, spaced 1 week apart, utilizing Robert Maigne's technique (a specific manual therapy protocol) as an adjunctive treatment alongside their physiotherapy.	3 months' follow-up (to measure the mid-term retention and stability of the clinical improvements)	Both groups experienced a statistically significant decrease in back and leg pain. However, between-group analyses showed significantly better outcomes for the manipulation group across all measurements with large effect sizes. Only the manipulation group demonstrated significantly favorable results on the ODI (P < 0.001) and the SLR test (P = 0.001). All spinal ROM increased significantly with manipulation (P < 0.001), whereas the control group only showed improvements in right/left rotation and extension.	Adding spinal manipulation therapy to a conventional physical therapy regimen significantly improves clinical outcomes and physical mobility over a 3-month period for individuals suffering from subacute or chronic lumbar radiculopathy.
Two manual therapy techniques for management of lumbar radiculopathy: a randomized clinical trial [20]	Danazumi et al., 2021	60	VAS: Used to evaluate both back pain and leg pain intensity. RMDQ: Used to assess back-pain-specific functional disability. SBI: Used to measure how irritating or disruptive the sciatica symptoms were to daily life.	Participants were randomly allocated into three equal groups (n = 20) each. Each group attended two 30-minute treatment sessions per week for 3 months: Group 1 (SMWLM): Received alone (a Mulligan concept technique designed to correct positional faults and relieve nerve compression). Group 2 (PINS): Received alone (a technique using progressive ischemic compression designed to normalize neuromuscular reflex activity). Group 3 (Combined): Received a simultaneous protocol combining both SMWLM and PINS.	Long-term tracking up to 9 months. Assessments occurred at baseline, immediately post-treatment (3 months), 3 months follow-up (6 months from start), 6 months follow-up, and 9 months follow-up.	A repeated-measures Analysis of Variance revealed significant interactions between the types of intervention and time for all outcome variables (P = 0.001). Patients in the combined group (SMWLM + PINS) experienced significantly greater improvements in leg pain, back pain, functional disability, and sciatica symptoms at every single assessment timeline compared to either technique used individually (P < 0.05). When comparing the standalone techniques, the SMWLM group demonstrated significantly better clinical outcomes than the PINS-alone group across all follow-up intervals (P < 0.05).	Combining SMWLM and PINS produces superior clinical outcomes. The combined protocol is highly recommended over utilizing either manual therapy technique on its own for managing individuals with lumbar radiculopathy.
Effects of non-surgical decompression therapy in addition to routine physical therapy on pain, range of motion, endurance, functional disability and quality of life versus routine physical therapy alone in patients with lumbar radiculopathy: a randomized controlled trial [21]	Amjad et al., 2022	60	Pain Intensity: Measured using a VAS at rest. Functional Disability: Evaluated via the Urdu version of the ODI. Lumbar ROM: Measured via the MMST for flexion and extension. BME: Evaluated using the prone isometric chest raise test. QOL: Measured across multiple health domains using the SF-36 Health Survey.	Control Group (Routine Physical Therapy): Received a conventional physiotherapy program including a hot pack, TENS, and targeted lumbar stretching and strengthening exercises. Experimental Group: Received the exact same routine physical therapy protocol plus NSD therapy using a computerized feedback mechanism for segmental distraction of the spinal nerve roots.	No extended long-term follow-up tracking was performed beyond the treatment period. Measurements were recorded at baseline (pre-treatment) and immediately post-intervention following the completion of the multi-week care protocol.	An analysis of covariance analysis showed a statistically significant between-group improvement (P < 0.05) in favor of the decompression (NSD) group across nearly all core variables. The NSD group achieved a significantly greater reduction in pain VAS (P < 0.001) and functional disability ODI (P < 0.001). The NSD group also achieved significantly greater gains in back muscle endurance (P = 0.002), lumbar flexion (P < 0.001), lumbar extension (P < 0.001), as well as the Role Physical (P = 0.019) and Bodily Pain (P = 0.016) domains of the SF-36 survey.	Combining computerized non-surgical spinal decompression therapy with conventional physical therapy is statistically and clinically superior to physical therapy alone. The integrated approach provides significantly better relief from pain while more effectively restoring lumbar mobility, trunk muscle endurance, daily functional capacity, and physical QOL in patients suffering from lumbar radiculopathy.
Comparison of the effects of conventional physiotherapy and proprioception exercises on pain and ankle proprioception in patients with lumbar radiculopathy [22]	Senol et al., 2022	119	Lumbar Pain Intensity: Assessed using the NPRS. Ankle Proprioception: Measured objectively using an isokinetic dynamometer to calculate target position repositioning errors (angular differences) at three distinct angles: 10˚ dorsiflexion, 11˚ plantarflexion, and 25˚ plantarflexion.	The 89 patients with lumbar radiculopathy were randomly assigned to one of three active treatment groups: Group 1 (n = 27): Received Conventional Physiotherapy alone. Group 2 (n = 31): Received Proprioception Exercises alone. Group 3 (n = 31): Received a combined protocol consisting of both Conventional Physiotherapy and Proprioception Exercises. Note: The 30 healthy volunteers in the control group did not receive any intervention.	No extended long-term tracking was performed beyond the active intervention phase. Assessments were recorded at baseline (pre-treatment) and immediately post-treatment to gauge the immediate therapeutic efficacy of the protocols.	Post-treatment evaluations revealed a statistically significant difference between the groups regarding both ankle proprioception angular errors and NPRS pain measurements (P < 0.05). Statistically significant improvements were noted when comparing the Conventional Therapy alone group to the Proprioception Exercises alone group, as well as comparing the combined Conventional Therapy and Proprioception Exercises group to the healthy controls. Importantly, there was no statistically significant difference when comparing the Conventional Therapy group directly against the Proprioception Exercises group, or when comparing the combined Conventional Therapy and proprioception exercises group against the healthy control group (P > 0.05). This indicates that the combination protocol managed to successfully normalize the patients' scores to the level of healthy individuals.	Patients with lumbar radiculopathy experience notable deficits in ankle joint position sense. Utilizing a combined regimen of conventional physiotherapy and targeted proprioception exercises is the most clinically effective method for simultaneously reducing lumbar pain and correcting ankle proprioception errors which are critical elements for restoring standard balance and motor coordination.
Efficacy of ultrasound versus short wave diathermy in the treatment of chronic low back pain in patients with lumbar disk herniation: a prospective randomized control study [23]	Ozen et al., 2023	93	Pain Intensity: Evaluated via a VAS specifically for low back pain. Functional Disability: Measured using the Modified ODI. QOL: Evaluated using the SF-36 Health Survey across physical, mental, and social health domains.	All participants received a baseline program consisting of 10 sessions of a hot pack (20 minutes), TENS at 100Hz (20 minutes), and a standard regimen of abdominal/lower back muscle strengthening exercises. Participants were split into three arms: Group 1 (Ultrasound): Received the baseline program plus 10 sessions of continuous deep heating therapeutic ultrasound (1 MHz, 1.5 W/cm², applied to the lower back for 10 minutes). Group 2 (Short Wave Diathermy): Received the baseline program plus 10 sessions of short wave diathermy using an induction technique (27.12 MHz, wavelength 11.06 meters, continuous thermic mode, applied to the lower back for 20 minutes). Group 3 (Control): Received the baseline program of hot packs, TENS, and therapeutic exercise alone.	Mid-term tracking up to 3 months. Assessments were explicitly recorded at four checkpoints: baseline (pre-treatment), immediately post-treatment (2 weeks), 1-month follow-up, and 3-month follow-up.	Pain and Functionality: All 3 groups demonstrated highly significant intra-group improvements in VAS pain scores and Modified ODI disability scores post-treatment and through the 1 and 3-month follow-ups (P < 0.001). Sustained Improvement: Only the deep heating groups (Group 1 and Group 2 Short wave diathermy demonstrated a continued, progressive reduction in functional disability (Modified ODI scores) moving from the immediate post-treatment mark into the 1 and 3-month follow-up phases (P < 0.001 and P = 0.012, respectively). QOL: While physical function, social function, and pain parameters on the SF-36 improved across all three groups (P < 0.05), the deep heating groups (ultrasound and short-wave diathermy) achieved significantly better psychological improvements, including role limitations due to physical/emotional issues, emotional well-being, vitality, and overall mental health (P < 0.05).	Deep heating physical modalities, specifically therapeutic ultrasound and short-wave diathermy, provide positive mid-term benefits when integrated into conservative rehabilitation protocols. While basic physical therapy (TENS, heat, exercise) successfully reduces pain initially, adding ultrasound or short-wave diathermy yields progressively superior, sustained improvements in physical functionality and mental QOL for patients managing chronic low back pain from lumbar disc herniations.
Effects of spinal manipulation or mobilization as an adjunct to neurodynamic mobilization for lumbar disc herniation with radiculopathy: a randomized clinical trial [24]	Danazumi et al., 2023	40	Primary outcomes (at 12 weeks): Back and leg pain intensity evaluated via the VAS, and activity limitations assessed via the RMDQ. Secondary outcomes: Sciatica bothersomeness via the SBI, sciatica frequency, functional mobility via the TUG test, health-related QOL via the SF-36 survey, and global perceived effect.	Group 1: Spinal Manipulative Therapy (high-velocity low-amplitude thrusts) combined with Neurodynamic Mobilization. Group 2: Spinal Mobilization (oscillatory/grade movements) combined with Neurodynamic Mobilization.	Long-term follow-up evaluated post-intervention	Both groups showed significant improvements, but the Spinal Mobilization group achieved statistically significant superior outcomes over the manipulation group across multiple metrics, displaying large calculated effect sizes (e.g., d = 1.06 for leg pain, d = 1.12 for RMDQ, and d = 1.18 for the TUG test).	Spinal mobilization combined with neurodynamic mobilization is clinically more effective than spinal manipulation as an adjunctive treatment for reducing pain, improving mobility, and lowering functional disability in patients experiencing lumbar disc herniation with radiculopathy.
Long-term effects of lumbar flexion versus extension exercises for chronic axial low back pain: a randomized controlled trial [25]	Park et al., 2024	56	Pain Metrics: Average back pain intensity (Primary), current pain, least pain, worst pain, and pain interference all quantified using a standard 0–10 rating scale. Functional and QOL Scales: ODI for back-specific function, EQ-5D for health-related QOL, and PASE.	Both groups completed a base protocol consisting of four supervised exercise education sessions with a physical therapist, followed by a strict requirement to perform their assigned exercises daily at home: Flexion Group: Performed a lumbar flexion-based exercise program (focused on flattening the lumbar curve and flexing the spine, e.g., William's flexion exercises). Extension Group: Performed a lumbar extension-based exercise program (focused on maintaining/restoring the lumbar lordosis and strengthening posterior structures, e.g., McKenzie-type extension exercises).	Long-term tracking for 1 year (12 months). Assessments were completed at baseline, 1 month, 3 months, 6 months, and 12 months.	Pain Relief: At the 1-year mark, the extension group achieved significantly lower average back pain scores compared to the flexion group, yielding a statistically significant between-group difference of 1.52 favoring extension (P = 0.002). Subscale trends: The extension group also demonstrated significantly greater long-term improvements in current pain, least pain, and overall pain interference (P < 0.05). No significant differences were seen between the groups regarding "worst pain". Functional Status: Functional limitations and QOL scales (ODI, EQ-5D, PASE) improved over time but remained statistically similar between the two treatment groups at the 1-year endpoint. Safety: Both exercise patterns were safe; no serious adverse events occurred, and mild, transient low back soreness was distributed evenly between both cohorts.	For individuals dealing with chronic axial low back pain, a home-based lumbar extension exercise regimen is significantly more effective at minimizing long-term average pain levels and reducing daily pain interference over a 1-year period than a flexion-based program. These findings strongly advocate for prioritizing lumbar extension movement patterns when designing therapeutic exercise plans for chronic axial back conditions.
Effect of thoracic spine mobilization on pain and lumbar mobility in patients with lumbosacral radiculopathy: a randomized controlled trial [26]	Rehab et al., 2024	34	Back Pain Intensity: Evaluated using the VAS for lower back localized pain. Radiated Leg Pain Intensity: Evaluated using the VAS tracking radiating sciatic symptoms down the leg. Lumbar Spine Mobility: Evaluated by measuring active spinal ROM.	Control Group (Conventional Physiotherapy): Received a baseline physical therapy program that included TENS at 4 Hz for 20 minutes, continuous therapeutic ultrasound (1 MHz, 0.5 W/cm²) for 5 minutes, and a targeted lumbar stabilization exercise protocol. Study/Experimental Group (Thoracic Mobilization + Conventional): Received the exact same conventional physical therapy program plus manual thoracic spine mobilization. The therapist applied Grade 2–3 central postero-anterior glides to the spinous processes of the T5–T12 vertebrae. The mobilization was performed for 60 seconds followed by a 15-second rest, repeated continuously for 5 minutes.	No long-term follow-up tracking was performed after the completion of care. Assessments were recorded at two distinct points: baseline (pre-treatment) and immediately post-treatment to analyze the direct therapeutic value of adding thoracic interventions.	Within-Group Changes: Both groups experienced highly significant improvements over time (P < 0.001). The study group saw a 49.56% reduction in back pain and a 42.94% reduction in radiated leg pain, while the control group only improved by 27.15% and 23.11%, respectively. Additionally, lumbar mobility increased by 16.95% in the study group compared to only 7.38% in the control group. Between-Group Changes: Post-treatment comparisons revealed a statistically significant difference (P < 0.01) across all outcome measures strongly favouring the study group. Patients who received thoracic mobilization alongside standard care achieved vastly superior pain relief and better lumbar mobility metrics.	Incorporating thoracic spine mobilization serves as a highly beneficial adjunct to standard physical therapy programs, yielding significantly better reductions in both back and radiating sciatic pain while markedly improving regional lumbar mobility for individuals managing chronic lumbosacral radiculopathy
Comparison of positional distraction with stabilisation exercises versus stabilisation exercises alone in the management of lumbar radiculopathy: a randomized controlled-trial [27]	Khan et al., 2024	100	Pain Intensity: Evaluated via a 0-10 cm VAS. Functional Disability: Assessed via the 0-24 point RMDQ.	Both groups received treatment 3 sessions per week for a total duration of 8 weeks: Group A (Positional Distraction + Stabilization): Received 15 minutes of manual positional distraction (lying on the unaffected side over a soft roll with hips flexed and upper trunk rotated to decompress the neuroforamen). They also performed positional distraction at home 2-3 times daily, paired with a multi-phase spinal stabilization exercise protocol. Group B (Stabilization Alone): Performed the exact same multi-phase spinal stabilization exercise protocol alone (core training including abdominal tuck-ins, straight leg raises, hook-lying marching, prone leg extensions, and progression to physio-ball and wall-slide exercises).	No extended long-term tracking was performed beyond the active intervention window. Assessments were recorded at two specific points: baseline and immediately post-intervention (at the end of the 8-week treatment period).	Within-Group: Both groups achieved highly statistically significant improvements in pain and disability scores from baseline to post-intervention (P < 0.001). Between-Group: Post-treatment data analysis showed that Group A (Positional Distraction + Stabilization) achieved significantly better outcomes for both VAS pain reduction and RMDQ functional disability improvement compared to Group B (P < 0.05).	Integrating manual positional distraction into a core stabilization exercise program yields superior therapeutic results compared to executing stabilization exercises alone. It is a highly effective clinical strategy for maximizing pain relief and minimizing daily functional restrictions in patients dealing with lumbar radiculopathy.
Effectiveness of Shacklock's neural mobilization for acute and sub-acute lumbar disc prolapsed: a randomized controlled trial [28]	Nahid et al., 2025	42	Pain and Behavioral Impact: Evaluated using the DPQ to track position-specific and task-specific pain intensity (e.g., pain at night, pain during twisting, pain while walking/bending). Functional Disability: Assessed via the ODI.	Both groups received treatment at a frequency of 4 sessions per week for a duration of 2 weeks (totaling 8 sessions). Both cohorts also received structural posture education for sitting and standing positions. Control Group: Received standard/usual physiotherapy care alone. Experimental Group: Received standard/usual physiotherapy care plus Shacklock's neurodynamic mobilization protocol (a manual therapy technique designed to mechanically mobilize nerve roots, manage neural tension, and restore sliding capacity).	No long-term follow-up tracking was performed beyond the active treatment period. Patient metrics were recorded at two points: baseline (pre-test) and immediately post-treatment (at the conclusion of the 2-week protocol).	Within-Group Changes: Both the experimental and the control groups experienced highly significant improvements in pain intensity and functional capacity from pre-test to post-test (P < 0.05). For example, the experimental group's median general pain/disability score dropped from 6.5 to 3.3. Between-Group Changes: Statistical analysis via independent sample t-tests demonstrated no statistically significant difference between the two groups at the post-treatment evaluation. The inclusion of Shacklock's mobilization did not result in clinically superior outcomes when compared directly against standard physiotherapy alone. Additionally, no statistically significant associations were discovered between post-treatment disability status and demographic factors like gender (P = 0.69) or Body Mass Index (P = 0.41).	Both standard physiotherapy and standard care supplemented with Shacklock's neural mobilization are effective methods for mitigating pain and disability in individuals with acute and sub-acute lumbar disc prolapse. However, because between-group metrics were statistically equivalent, adding Shacklock's neural mobilization does not yield superior clinical outcomes over a basic, well-structured conventional physical therapy program alone during a 2-week care plan.
A comparative analysis of neural mobilization techniques and conventional physical therapy for sciatica pain management in lumbar radiculopathy [29]	Arshad et al., 2025	60	Primary outcome: Pain intensity evaluated via the VAS. Secondary outcomes: Functional disability assessed using the ODI and lumbar ROM tested via the SLR test.	NMT Group (n = 30): Received targeted NMT specifically focusing on sciatic nerve "slider" and "tensioner" maneuvers. CPT Group (n = 30): Received CPT consisting of a standardized protocol of lumbar stabilization exercises, spinal stretching, and basic electrotherapy.	Short-term tracking up to 8 weeks. Assessments were recorded at three specific milestones: baseline, 4 weeks post-intervention, and 8 weeks post-intervention.	Within-Group: Both treatment approaches yielded statistically significant improvements across all pain, disability, and ROM metrics over time (P < 0.05). Between-Group: The neural mobilization cohort demonstrated vastly superior therapeutic improvements. At the final 8-week assessment, the NMT group showed significantly lower pain scores (Mean Difference: -2.4; P < 0.001) and significantly reduced disability levels (Mean Difference: -12.8; P < 0.001) compared to the CPT group. SLR mobility was also significantly higher in the NMT group.	Neural mobilization techniques (sliders and tensioners) are significantly more effective at alleviating neuropathic pain, reducing daily functional disability, and restoring lower limb mobility than traditional physical therapy modalities alone for patients struggling with sciatica secondary to lumbar radiculopathy.
Short-term effects of manual therapy combined with functional magnetic stimulation in individuals with lumbar disk herniation with radiculopathy: a randomized clinical trial [30]	Lytras D et al., 2026	40	Pain Intensity: NPRS assessed for both lumbar and leg pain. Functional Disability: RMDQ. Neuropathic Pain Symptoms: S-LANSS. Neural mechanosensitivity: SLR, ROM.	Participants were randomly allocated into two equal groups 1. Manual Therapy + Functional Magnetic Stimulation Group (n = 20): Received Manual Therapy combined with Functional Magnetic Stimulation. 2. Manual Therapy-Only Group (n = 20): Received Manual Therapy alone. Both groups received a total of 10 treatment sessions administered over a 3-week period.	3 weeks (assessed at baseline and immediately post-intervention at week 3).	A two-way mixed analysis of variance demonstrated statistically significant group time interactions favoring the combined group across all parameters: Interactions: Significant for all outcomes (p < 0.01), showing greater statistical and clinical improvements in the MT + FMS group. Clinical relevance: Reductions in lumbar pain, leg pain, functional disability, and S-LANSS scores comfortably exceeded MCID. Threshold Neural Mobility: SLR range of motion gains surpassed the Minimal Detectable Change value. Neuropathic Resolution: Only the MT + FMS group saw average symptoms drop below the official diagnostic cutoff for neuropathic pain (S-LANSS score less than 12).	Incorporating functional magnetic stimulation alongside a manual therapy protocol provides superior short-term clinical benefits compared to manual therapy alone. The dual approach effectively reduces pain intensity, minimizes functional disability, reduces neural mechanosensitivity, and mitigates neuropathic pain features in individuals suffering from chronic lumbar disk herniation with radiculopathy.

Assessment of Risk of Bias

The risk of bias assessment using the RoB 2 tool across the included RCTs indicated a variable methodological quality; while some studies exhibited a low risk of bias, others raised some concerns or were classified as having a high risk of bias. Most studies demonstrated a low risk of bias in key domains such as classification of interventions, missing outcome data, and measurement of outcomes, suggesting the use of appropriate assessment tools like Visual Analogue Scale (VAS) and Oswestry Disability Index (ODI) and generally complete reporting of results. However, a consistent source of concern across several trials was bias due to deviations from intended interventions, largely reflecting challenges in blinding participants and therapists in physiotherapy-based manual therapy studies. A moderate risk of bias was also observed in some studies related to the randomization process, selection of participants, and selective reporting, particularly in trials with less rigorous protocol registration or unclear allocation procedures. Table 2 illustrates the risk of bias assessment of the included 19 studies.

**Table 2:** Risk of bias assessment of randomized intervention using ROB 2 tool

Study (Year)	Bias arising from the randomization process	Bias due to deviations from intended interventions	Bias due to missing outcome data	Bias in the measurement of the outcome	Bias in the selection of the reported result	Overall Risk of Bias
Bello et al. (2019) [12]	Low Risk	Some Concerns	Low Risk	Low Risk	Low Risk	Some Concerns
Satpute et al. (2019) [13]	Low Risk	Some Concerns	Low Risk	Low Risk	Low Risk	Some Concerns
França et al. (2019) [14]	Low Risk	Some Concerns	Low Risk	Low Risk	Low Risk	Some Concerns
Plaza-Manzano et al. (2020) [15]	Low Risk	Some Concerns	Low risk	Low Risk	Low risk	Some Concerns
Kostadinović et al. (2020) [16]	Low Risk	Some Concerns	Low Risk	Low Risk	Low Risk	Some Concerns
Sanei et al. (2020) [17]	Low Risk	High Risk	Low Risk	Some Concerns	Low Risk	High Risk
Munjupong and Kumnerddee (2020) [18]	Low Risk	Low Risk	Low Risk	Low Risk	Low Risk	Low Risk
Ghasabmahaleh et al. (2021) [19]	Low Risk	High Risk	Low Risk	Some Concerns	Low Risk	High Risk
Danazumi et al. (2021) [20]	Low Risk	Some Concerns	Low Risk	Low Risk	Low Risk	Some Concerns
Amjad et al. (2022) [21]	Low Risk	High Risk	Low Risk	Some Concerns	Low Risk	High Risk
Senol et al. (2022) [22]	Low Risk	High Risk	Low Risk	Some Concerns	Low Risk	Some Concerns
Ozen et al. (2023) [23]	Low Risk	Some Concerns	Low Risk	Low Risk	Low Risk	Some Concerns
Danazumi et al. (2023) [24]	Low Risk	Some Concerns	Low Risk	Low Risk	Low Risk	Some Concerns
Park et al. (2024) [25]	Low Risk	Some Concerns	Low Risk	Low Risk	Low Risk	Some Concerns
Rehab et al. (2024) [26]	Low Risk	Some Concerns	Low Risk	Some Concerns	Low Risk	Some Concerns
Khan et al. (2024) [27]	Low Risk	High Risk	Low Risk	High Risk	Low Risk	High Risk
Nahid et al. (2025) [28]	Low Risk	High Risk	Low Risk	High Risk	High Risk	High Risk
Arshad et al. (2025) [29]	Low Risk	High Risk	Low Risk	High Risk	Low Risk	High Risk
Lytras D et al. (2026) [30]	Low Risk	Some Concerns	Low Risk	Low Risk	Low Risk	Some Concerns

Discussion

This systematic review synthesizes current evidence supporting conservative treatment strategies for the management of lumbar radiculopathy across diverse clinical settings. The included literature primarily involved middle-aged individuals with subacute to chronic presentations, most commonly secondary to lumbar disc herniation. These patients were characterized by radiating lower-limb pain, neurological symptoms, functional limitations, and reduced quality of life [1]. Across the reviewed studies, a broad spectrum of conservative interventions demonstrated clinically meaningful benefits, including exercise-based rehabilitation, spinal mobilization and manipulation, neural mobilization, trunk stabilization, and directional preference strategies. Collectively, these evidence-based conservative interventions were associated with significant reductions in pain and disability, alongside improvements in muscular strength and functional performance [2]. These outcomes were most frequently captured using validated tools such as the VAS, Numerical Pain Rating Scale (NPRS), and ODI [1-3].

When analyzing specific manual therapy protocols, the evidence reveals a critical distinction between the efficacy of isolated techniques versus multimodal care. For instance, Bello et al. compared posteroanterior intermittent nucleus mobilization (PINS) against SMWLM, finding significant within-group improvements but no significant between-group differences in pain or disability at eight weeks [12]. This null comparative finding suggests that individual manual therapies may yield equivalent outcomes when applied in isolation. Conversely, Ghasabmahaleh et al. demonstrated that adding three sessions of Maigne's spinal manipulation to a baseline physiotherapy program resulted in significantly greater improvements in disability, ROM, and functional outcomes compared to physiotherapy alone [19]. Lytras et al. (2026) discuss how adding functional magnetic stimulation (FMS) to manual therapy significantly enhances short-term clinical outcomes by targeting both mechanical and neuropathic pain pathways in patients with lumbar disk herniation with radiculopathy. The authors emphasize that the deep magnetic pulses of FMS help down-regulate neural mechanosensitivity and accelerate functional recovery, successfully driving neuropathic symptoms below diagnostic thresholds. Ultimately, they conclude that this combined, non-invasive approach provides a superior, rapid-acting therapeutic synergy compared to using manual therapy alone [30].

However, the integration of specialized modalities requires careful, outcome-specific nuance rather than broad assumptions of superiority. The randomized trial by Plaza-Manzano et al. provides a critical test case; they found that adding neurodynamic mobilization to a motor control exercise program failed to produce superior outcomes for global pain and disability when compared to motor control exercises alone [15]. While this represents a meaningful negative result for primary clinical markers, the neurodynamic group did exhibit significantly greater improvements in specific neuropathic symptom severity and SLR performance [15]. This highlights that neurodynamic techniques should not be viewed as generalized tools to amplify overall pain reduction, but rather as highly targeted adjuncts designed specifically to restore neural mobility and reduce mechanosensitivity. Consequently, conflicting evidence in the literature can largely be reconciled by distinguishing between global symptomatic relief and targeted physiological adaptations.

A notable omission across the majority of the reviewed literature is the systematic reporting of adverse events and safety data. While conventional manual therapy and exercise are widely regarded as safe first-line interventions for lumbar radiculopathy, specific data tracking transient symptom exacerbation, increased radicular irritation, or treatment intolerance were generally absent or underreported in the primary trials. Only minor, self-limiting soreness was occasionally noted, and no major neurovascular complications or progressions to cauda equina syndrome were recorded. This lack of rigorous safety reporting limits the ability to construct a definitive risk-benefit profile for these mechanical interventions, emphasizing the need for standardized safety tracking in conservative radiculopathy trials.

Clinical Implications

The discoveries of this systematic review strongly support conventional therapy as the initial course of treatment for lumbar radiculopathy. Clinically, interventions such as neural mobilization, spinal manipulation, SMWLM, motor control training, and stabilization exercises demonstrate meaningful improvements in pain, disability, and functional capacity. Neural mobilization appears particularly effective in reducing nerve mechanosensitivity and radicular pain, while spinal manipulation and mobilization techniques enhance short- to mid-term functional recovery [14,29].

Importantly, multimodal rehabilitation combining manual therapy with exercise therapy yields superior outcomes compared to single interventions. Clinicians should therefore adopt an individualized, impairment-based approach, integrating neurodynamic techniques, spinal manual therapy, and core stabilization exercises based on patient presentation and chronicity of symptoms. Early conservative intervention may also reduce progression to chronic disability and decrease the need for surgical management.

Limitations

This systematic review has several limitations that should be considered. A major limitation is the substantial clinical heterogeneity across included studies. Using the PICOT framework, variability was observed in population characteristics, including age, symptom duration, severity, and diagnostic criteria for lumbar radiculopathy. Gray literature was excluded to ensure methodological consistency and availability of complete outcome data; however, this may have increased the risk of publication bias given the limited number of included studies. Formal assessment of publication bias (e.g., funnel plots or Egger’s test) was not performed because the number of studies was insufficient for reliable analysis. Accordingly, potential overrepresentation of positive findings cannot be ruled out.

Considerable heterogeneity was also present in interventions, with wide variation in manual therapy techniques, exercise protocols, treatment dosage, frequency, and duration. Comparators differed across studies, limiting direct comparisons, while outcome measures varied in focus and assessment tools, restricting synthesis of results. Additionally, most studies reported short-term follow-up, limiting conclusions regarding long-term effectiveness. Formal grading of evidence certainty using the Grading of Recommendations, Assessment, Development, and Evaluation (GRADE) framework was not undertaken due to pronounced heterogeneity among included studies, which limited the validity of pooled certainty judgments.

Methodological limitations were noted, with several trials demonstrating moderate risk of bias, particularly related to blinding and allocation concealment. Small sample sizes further reduced statistical power and generalizability. Overall, these limitations highlight the need for future well-designed trials with standardized populations, interventions, outcomes, and follow-up durations to strengthen the evidence base.

Future Scope

To address the current gaps identified in the literature, future research initiatives must prioritize large-scale, high-powered, multicentric RCTs utilizing strictly standardized treatment protocols and uniform diagnostic criteria that integrate clinical, neurological, and advanced imaging assessments. Furthermore, implementing longitudinal tracking protocols with follow-up durations of at least six to 12 months is essential to evaluate the long-term durability of conservative interventions and accurately monitor symptom recurrence rates. Future studies should also systematically examine the dose-response relationships and optimal parameter configurations of manual therapy and neurodynamic techniques to help establish definitive clinical guidelines. Additionally, there is a critical need for subgroup and stratification analyses to explore personalized rehabilitation trajectories by dividing cohorts based on explicit clinical phenotypes, such as acute versus chronic symptom duration, specific features of disc pathology, and the presence or absence of neuropathic pain characteristics. Finally, future trials must incorporate the rigorous, standardized reporting of adverse events to build robust safety profiles, alongside comprehensive cost-effectiveness and health economic analyses to justify the widespread integration of multimodal physiotherapy within public and private healthcare delivery models.

## Conclusions

This systematic review summarizes recent clinical trial evidence on conservative interventions for lumbar radiculopathy. While several conservative approaches were associated with improvements in pain and function, interpretation is limited by substantial clinical and methodological heterogeneity and common trial-level limitations, including incomplete blinding.

Accordingly, the evidence should be interpreted cautiously, and firm conclusions regarding comparative effectiveness cannot be drawn. The findings instead indicate emerging trends that support the need for well-designed, adequately powered randomized controlled trials with standardized diagnostic criteria, intervention protocols, outcome measures, and adverse event reporting.

## Appendices

PubMed / MEDLINE

("lumbar radiculopathy"[Title/Abstract]  OR "lumbosacral radiculopathy"[Title/Abstract]  OR "lumbar nerve root compression"[Title/Abstract]  OR "sciatica"[Title/Abstract] OR "lumbar disc herniation with radiculopathy"[Title/Abstract]) AND ("clinical diagnosis"[Title/Abstract]  OR "diagnostic tests"[Title/Abstract]  OR "diagnosis"[MeSH Terms] OR "clinical examination"[Title/Abstract] OR "neurological examination"[Title/Abstract] OR "special tests"[Title/Abstract] OR "straight leg raise"[Title/Abstract] OR "slump test"[Title/Abstract] OR "imaging"[Title/Abstract] OR "radiography"[Title/Abstract]  OR "radiography"[MeSH Terms] OR "MRI"[Title/Abstract]"[MeSH Terms])AND English[Language] NOT ("animal"[MeSH Terms] NOT "human"[MeSH Terms])NOT ("artificial intelligence"[Title/Abstract])

Web of Science

TS= ("lumbar radiculopathy"  OR "lumbosacral radiculopathy"  OR "lumbar nerve root compression" OR "sciatica" OR "lumbar disc herniation with radiculopathy")AND TS=("clinical diagnosis" OR "diagnostic tests" OR "clinical examination" OR "neurological examination" OR "special tests" OR "straight leg raise" OR "slump test" OR "imaging" OR "radiography"  OR "MRI” TS=("artificial intelligence" OR "machine learning" OR "AI")AND LA=(English)

Scopus

TITLE-ABS-KEY("lumbar radiculopathy" OR "lumbosacral radiculopathy" OR "lumbar nerve root compression"  OR "sciatica" OR "lumbar disc herniation with radiculopathy")AND TITLE-ABS-KEY("clinical diagnosis" OR "diagnostic tests" OR "clinical examination"  OR "neurological examination "OR "special tests"  OR "straight leg raise" OR "slump test" OR "imaging" OR "radiography" OR "MRI"AND NOT TITLE-ABS-KEY("artificial intelligence" OR "deep learning" OR "machine learning")AND LIMIT-TO(LANGUAGE, "English")AND LIMIT-TO(EXACTKEYWORD, "Human")
